# Finite Element Analysis and Clinical Applications of Transverse Post for the Rehabilitation of Endodontically Treated Teeth

**DOI:** 10.7759/cureus.63719

**Published:** 2024-07-03

**Authors:** Shruthi Rajagopal, Sonali Sharma

**Affiliations:** 1 Department of Conservative Dentistry and Endodontics, Saveetha Dental College and Hospitals,Saveetha Institute of Medical and Technical Sciences (SIMATS) Saveetha University, Chennai, IND

**Keywords:** fiber post, post-endodontic restoration, non-vital tooth, conservative therapy, composite restoration

## Abstract

Introduction

The preservation of tooth structure during cavity preparation is crucial for maintaining tooth strength and longevity of restorations. The biomechanical behavior of teeth, especially those with mesio-occlusal-distal (MOD) cavity preparations, is significantly affected by the extent of cavity preparation and the type of restorative treatment employed. The aim of the current study was to evaluate and analyze the stress distribution seen in the mandibular molar with MOD cavity when restored with transverse post, using finite element analysis (FEA).

Materials and methodology

FEA was utilized to evaluate stress distribution in an endodontically treated mandibular first molar with MOD cavity preparation, restored using a transverse post and composite restoration. Three-dimensional models incorporating the tooth and the surrounding structures, along with the transverse post and composite restoration, were constructed based on known biomechanical properties. After meshing the models, loads were defined on the buccal and lingual cusps with a constant value of 600N and at an angle of 45 degrees. Preprocessing involved model preparation followed by postprocessing to obtain results representing the degree and type of stress distribution.

Results

FEA simulations revealed the distribution of stress within the tooth structure under functional occlusal forces. The transverse post system effectively reinforced the tooth by deflecting incident forces and providing uniform stress distribution. von Mises stresses were analyzed to assess the likelihood of material failure. The distribution of the stress in the restored tooth model was comparable to that seen in the intact model.

Conclusion

Transverse post along with composite restoration provides a conservative and cost-effective alternative to full coverage crowns while providing a functional and aesthetic outcome. Further research and clinical studies are warranted to validate these findings and optimize the clinical application of transverse post systems in restorative dentistry.

## Introduction

Evidence-based studies have demonstrated that the removal of tooth structure that is instrumental for caries excavation weakens the teeth and diminishes the strength [1]. As the number of involved walls increases, like in mesio-occlusal-distal (MOD) cavity preparations, the role of the restorative treatment becomes pivotal in reinforcing the remaining available tooth structure for the longevity of both the restoration and the teeth. The extent of the cavity and the type of restorative treatment utilized can influence the likelihood of crack propagation eventually leading to tooth fractures [2]. Joynt et al. conducted a study in 1987 which revealed that preparing an occlusal cavity leads to a 20% decrease in tooth stiffness. If the preparation also involves removing a marginal ridge, thereby converting the class I to class II cavity, the reduction in tooth stiffness is further exacerbated by 2.5 times, resulting in an overall 46% decrease in stiffness [3]. When both marginal ridges were included in the cavity preparation design, the stiffness diminished by 63%. Posterior teeth, especially maxillary premolars, have an anatomical shape that increases the likelihood of cusp fracture under occlusal loading [4].

The investigation of different approaches to restoring teeth, particularly focusing on class II conventional and MOD cavities, has been heavily carried on as the long-term survival of such restorations is questionable and could lead to catastrophic failures [5]. Such restorations have to endure heavy occlusal forces and greater amounts of lateral forces during mastication, as explained by the "C factor" concept [6]. Thus, the clinical evaluation of the remaining walls along with other factors of cavity preparation becomes critical in predicting the clinical outcomes of the restoration. Some of the key factors in cavity preparation are the depth of the cavity, width of the isthmus, cuspal reduction, and rounding of the remaining walls to accept the restoration of choice. The depth of the cavity has the greatest influence on the amount of stress within the tooth structure, due to an inverse relationship with the remaining dentin thickness (RDT). The RDT of a cavity not only predicts the longevity of the restoration but also provides pulpal protection against the insults from both the restoration material and the functional forces of occlusion [7]. It has been suggested that increasing the isthmus width involves more loss of tooth structure leading to greater stress on the restoration. Contrary to that, one study argues that it alters the cavity geometry in a manner that causes a favorable distribution of stress [8]. Additionally, certain studies have found no discernible impact of the isthmus width on the clinical longevity of the restored teeth.

The technique and type of restorative material used have a major bearing on the longevity of such restorations. It has been confirmed through evidence-based research that resin composite restorations have superior fatigue resistance than ceramic restorations. When comparing direct and indirect restorations, indirect restorations necessitate the removal of greater tooth structure than that required for direct restorations. Regarding amalgam cavity preparation, the circumstances are the same. With recent advances, bonded restorations utilizing various adhesive strategies have become the forefront in the functional replacement of missing tooth structure, promising favorable results in terms of the clinical longevity of the same [9]. According to Kuijs et al., for restorations involving cuspal replacement, the fatigue resistance offered by direct resin composite and indirect ceramic restorations are the same [10]. Conversely, Soares et al. discovered that cavities involving MOD preparations, when treated using direct composite placement, achieved superior biomechanical performance compared to those restored using indirect ceramic or resin materials [11]. However, in order to have more predictable clinical success, evaluation of stress distribution to the various parts of the tooth and their behavior has to be carried out in order to analyze different biomechanical properties of the tooth and restoration [12]. The vitality of the tooth also plays a role in the same. Non-vital teeth become brittle, due to changes in the physical and chemical properties of the tooth structure, like loss of water content [13].

Keeping these various clinical and operative factors in mind, it is imperative to study the stress analysis under various scenarios in order to make an informed evidence-based treatment plan. Finite element analysis (FEA) is a widely used method for studying dental biomechanics by dividing the geometry into small elements with known mechanical properties. Based on the concept of "moving from part to whole," this approach breaks the topic under investigation into digestible sections [14]. The three main steps of FEA are preprocessing, which prepares the modeling data, processing of the assembly that solves the equations, and postprocessing, which visualizes the findings after analysis. The rheological and physical characteristics of biological tissues and biomaterials are used to construct a model. To be more precise, figures pertaining to the modulus of elasticity, stress, and Poisson's ratio of the material are required for the final construction [15]. Post and core systems are regularly used as a way of reinforcing lost tooth structure. The novel idea of transfixing horizontal post systems in the buccal and lingual walls of MOD cavities has been suggested in order to conservatively reinforce the tooth and eliminate the need for full-crown coverage. Apart from being functional, it is a cheaper and less time-consuming method of producing decent aesthetic outcomes. Thus, a canopy of the fiber post was used in order to deflect incident forces and provide uniform stress distribution [16]. 

Therefore, the aim of the present study was to use three-dimensional finite element stress analysis to assess the distribution of stress when an endodontically treated mandibular first molar with MOD preparation is subjected to functional occlusal forces when restored with transverse fiber post and composite restoration. 

## Materials and methods

The output of FEA is expressed using various stresses, such as tensile, compressive, shear, or a combination known as von Mises stresses. When a complex loading condition is applied to an isotropic and ductile material, the von Mises stress is typically employed to determine if the material will yield. Principle stress, on the other hand, is the highest and lowest normal stress on a principle plane when there is no shear stress applied to a body. Though it is frequently inaccurate for ductile materials, the maximum primary stress theory is more reliable in predicting failure, particularly in brittle materials. Thus, von Mises stresses were taken into account in this investigation [17]. Based on the condition that all the parts are connected to one another by the nodes, it is possible to estimate the stresses, the variables that develop, and the overall deformation produced in each node. Because teeth are asymmetrical, reliable research requires the three-dimensional reproduction of the alveolar bone, periodontal ligament, and numerous other essential components to simulate the oral cavity [18]. 

**Figure 1:**
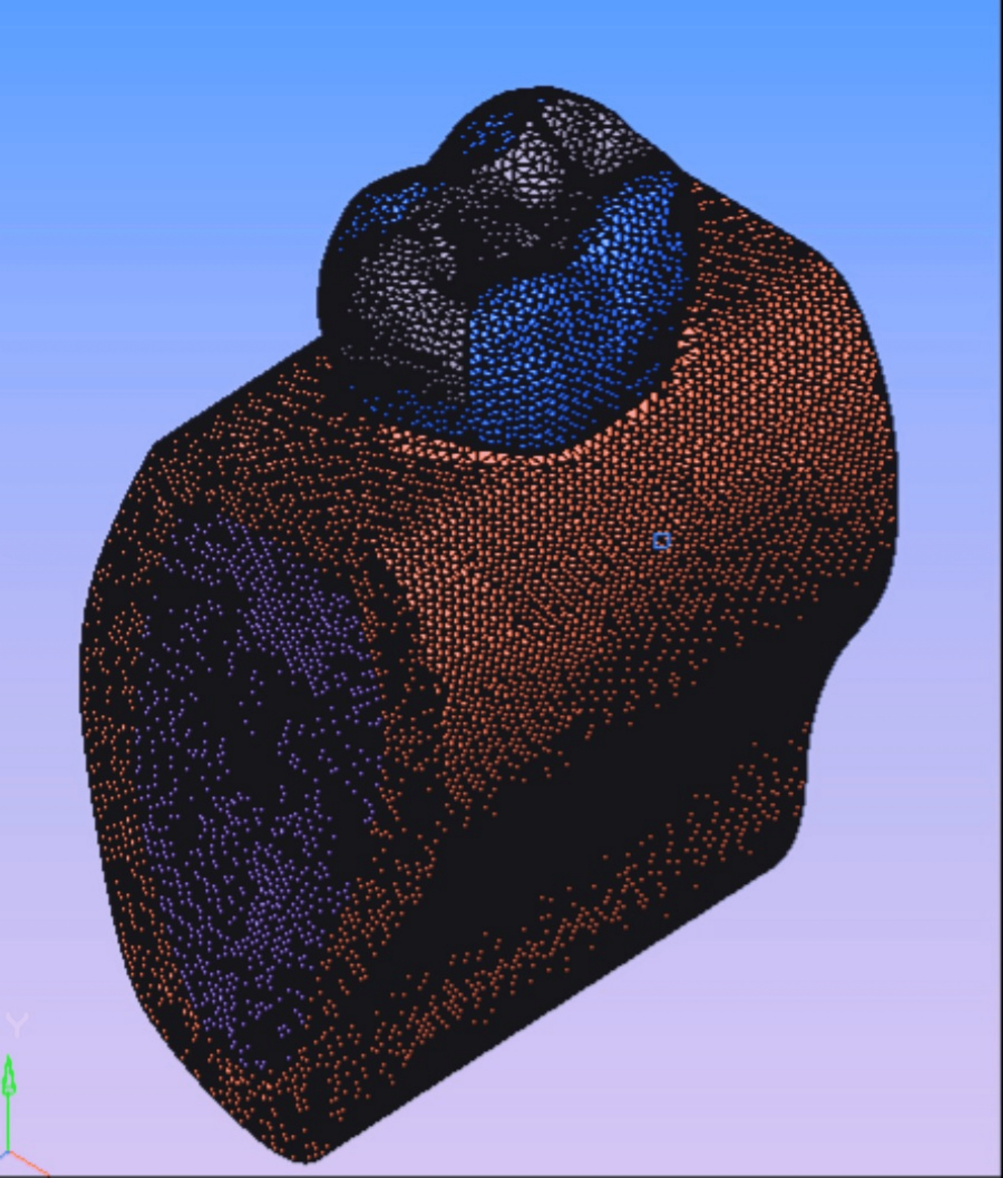
Model 1 representing the intact permanent mandibular molar and periapical tissue computed obtained using the elastic properties of individual structures after meshing

A three-dimensional model of the permanent mandibular first molar along with the periapical tissues and bone was made using the Ansys software (Ansys Inc., Canonsburg, Pennsylvania, United States). It was based on published data on material and physical qualities, as well as on the standard anatomy given in Wheeler's Dental Anatomy, Physiology and Occlusion [19]. The computer-simulated tooth model was composed of separate components connected at nodes, which were formed from the material properties. Meshing was done after the model was constructed and boundary conditions were established to ensure that the body under assessment was confined during the application of stress. The mandible and the first mandibular molar were the first parts of the model to be created. This is represented in Figure 1.

These models were designed on the Ansys software to represent circumstances similar to the oral cavity and are simulated and built using evidence-based scientific data to estimate the mechanical properties of materials, such as Poisson's ratio and elastic modulus of various structures as represented in Table 1. The models are based on extracted teeth but modeled and designed on the software.

**Table 1:** The elastic properties of the dental and bone tissues PDL: periodontal ligament

Materials	Elastic modulus (GPa)	Poisson's ratio
Enamel	93	0.3
Dentin	18.6	0.31
Pulp	0.002	0.45
PDL	0.0689	0.45
Alveolar bone	11.5	0.3
Cortical bone	13.7	0.3

**Figure 2:**
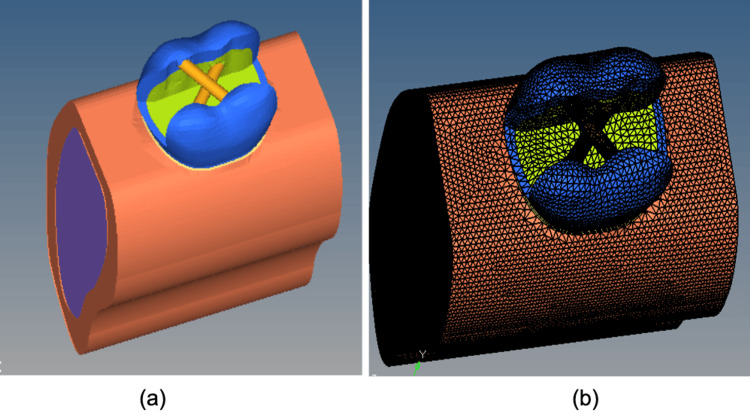
Model 2 depicting the restoration of the MOD cavity in a mandibular molar with transverse fiber post (a) before meshing and (b) after meshing MOD: mesio-occlusal-distal

It was assumed that all materials were linearly elastic, homogenous, and isotropic [20]. The models are three-dimensional models designed on the software, with no physical entity. As represented, these are graphic three-dimensional designs for the purpose of subjecting loading conditions that will help to evaluate the stress patterns. The following were the models: Model 1 is an exact replica of an intact first mandibular molar as represented in Figure 1. In Model 2, the subtraction Boolean method was used to recreate the prepared MOD cavity, leaving only the buccal and lingual walls intact. Transverse posts were inserted into the preparation 1.5 mm from the central buccal fissure, which has a diameter of 2 mm. This is represented in Figure 2.

In order to simulate the endodontic treated teeth, gutta-percha material was utilized. Two-thirds of the intercuspal distance was represented by the buccal-lingual wall distance. The adhesive protocol consisted of luting the post with resin cement (Panavia 2.0, Kuraray Dental Inc., Japan) and applying self-etch primer (Clearfil SE Bond, Kuraray Dental Inc., Japan). This was followed by restoration using the physical properties of a nanohybrid composite resin filling (Beautifil II, Shofu Dental Inc., Japan). A two-dimensional mesh was transformed into a three-dimensional mesh using the hypermesh drag options. With 10 nodes, the element type was tetrahedral. The models were meshes, boundary conditions were created, and the load was measured on the buccal and lingual distal cusps of the occlusal surface at a 45° angle and a constant axial load value of 600N after the application of a boundary condition. The next step was to do a three-dimensional FEA using the Ansys software.

Using the software, the stress patterns and extent of displacement under load were analyzed and recorded for both the models: Model 1 (replication of intact tooth) and Model 2 (replication of permanent non-vital mandibular molar with MOD cavity restored with transverse fiber post and composite resin cement).

## Results

**Figure 3:**
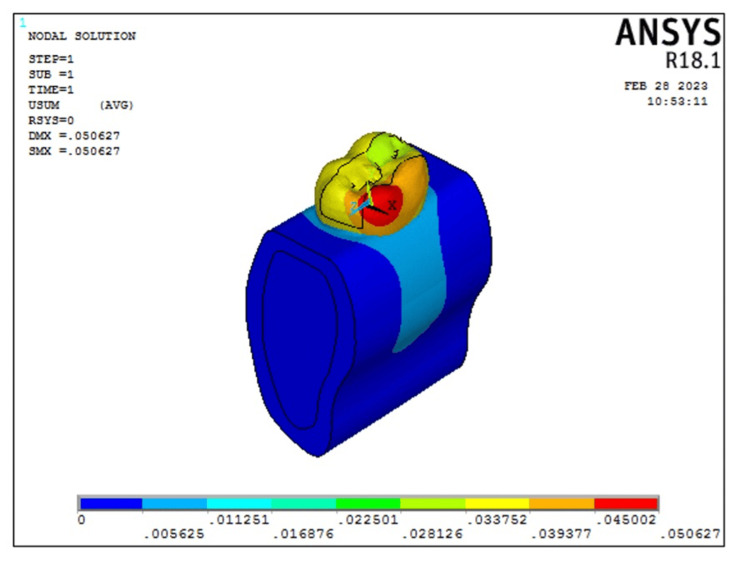
Overall deformation and von Mises stress distribution seen in Model 2 representing non-vital teeth with MOD cavity restored with fiber post and composite restoration. The color bar is used to visualize the results of structural analysis by representing the intensity of mechanical stress, with warmer colors indicating high-stress areas and cooler colors indicating low-stress areas MOD: mesial-occlusal-distal

The results of the FEA are represented as von Mises stresses and principle stresses. The following is a breakdown of the overall von Mises stress: Model 2 (transverse fiber post model) showed 119.294 MPa, whereas Model 1 (intact model) showed 154.83 MPa. After analyzing the overall deformation, it was found that the transverse fiber post group had a deformation of 0.050627 and the intact model had a deformation of 0.046879. For each model, a stress analysis was completed. Cortical bone stress was lower in Model 1 (intact model) which was 17.06 MPa as compared to Model 2 (transverse fiber post model) which was 7.7028 MPa. But in the cancellous bone, the stress was higher in Model 1 (intact model) which was 9.66112 MPa as compared to that in Model 2 (transverse fiber post model) which was 8.96254 MPa. The overall stress distribution is shown in Figure 3 for Model 2 representing the tooth restored with transverse fiber post.

**Figure 4:**
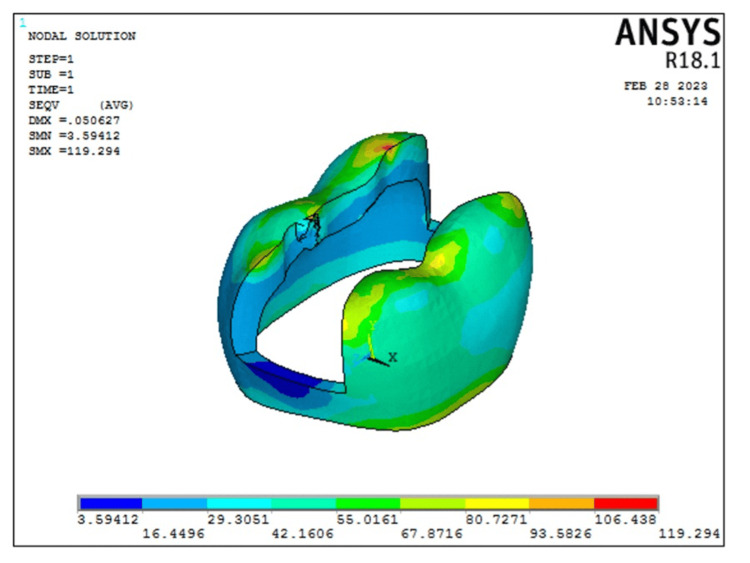
von Mises stress distribution as seen in enamel for Model 2 representing restoration with transverse fiber post model. The color bar is used to visualize the results of structural analysis by representing the intensity of mechanical stress, with warmer colors indicating high-stress areas and cooler colors indicating low-stress areas

According to the FEA, enamel, dentin, and pulp stress was higher in Model 1 representing intact tooth (154.83 MPa, 80.7399 MPa, and 0.000000542 MPa, respectively) as compared to Model 2 representing transverse fiber post model (119.294 MPa, 78.0512 MPa, and 0.000000525 MPa, respectively). The stress distribution in enamel as seen in Model 2 is represented in Figure 4.

**Figure 5:**
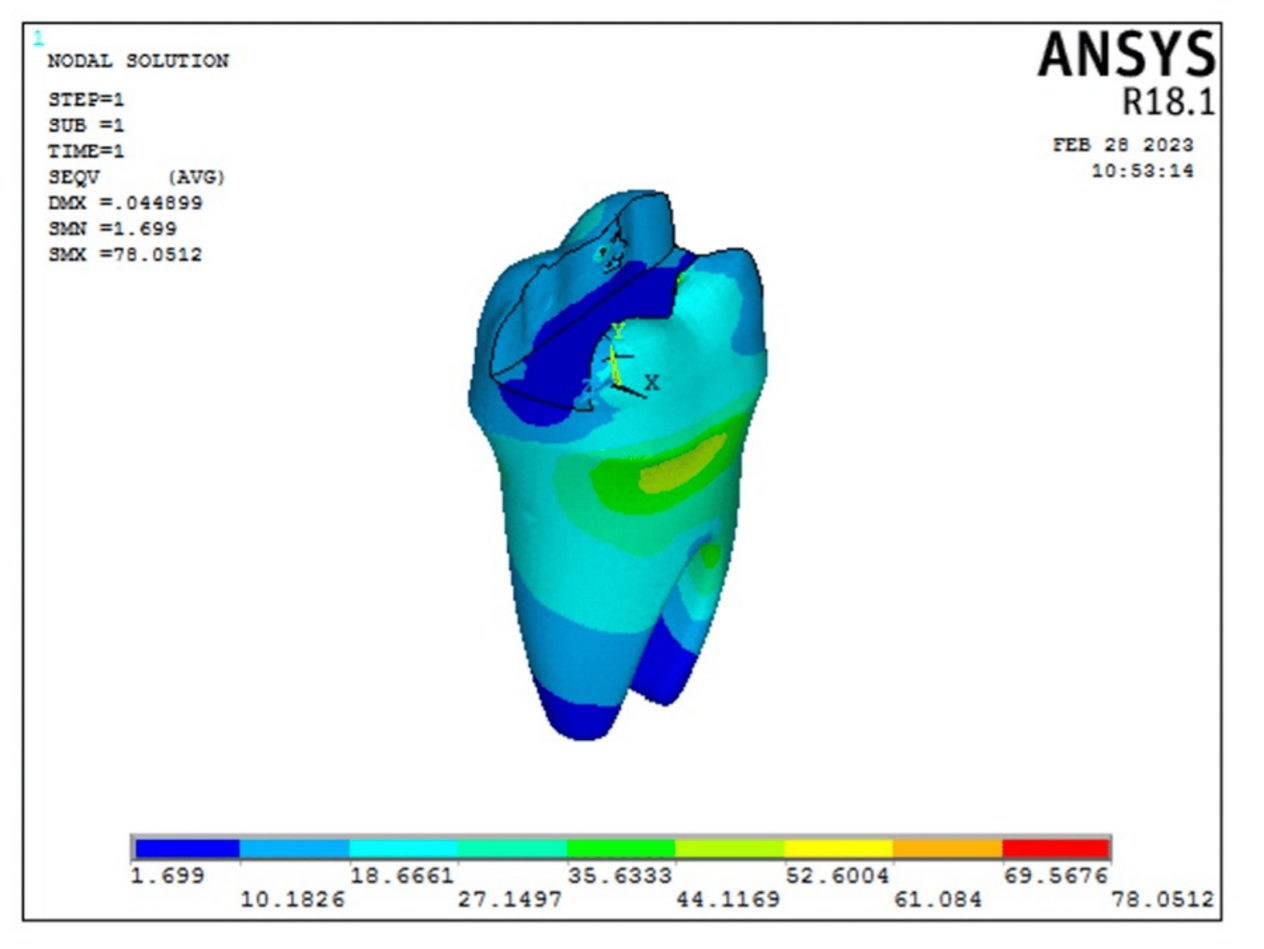
von Mises stress distribution as seen in dentin in Model 2 representing restoration with transverse fiber post model. The color bar is used to visualize the results of structural analysis by representing the intensity of mechanical stress, with warmer colors indicating high-stress areas and cooler colors indicating low-stress areas

The stress distribution in dentin as seen in Model 2 is represented in Figure 5.

**Figure 6:**
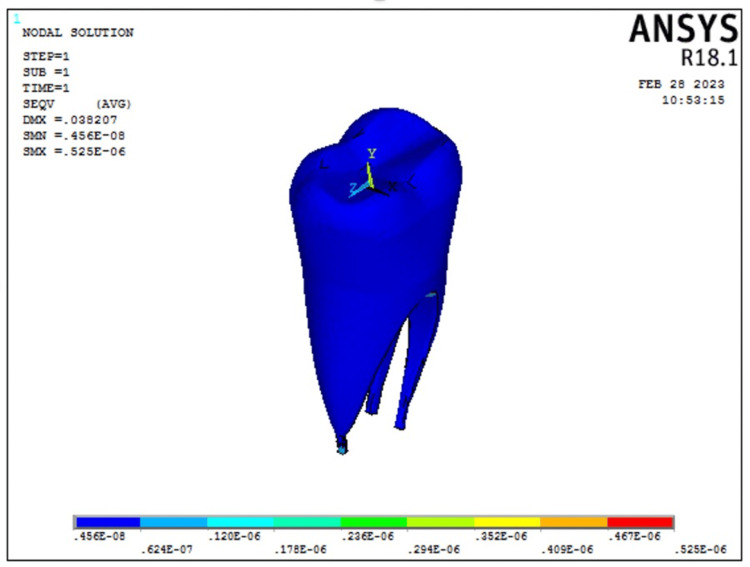
von Mises stress on the pulp as seen in Model 2 representing restoration with transverse fiber post model. The color bar is used to visualize the results of structural analysis by representing the intensity of mechanical stress, with warmer colors indicating high-stress areas and cooler colors indicating low-stress areas

The stress distribution on the pulp as seen in Model 2 (transverse fiber post model) is represented in Figure 6.

**Figure 7:**
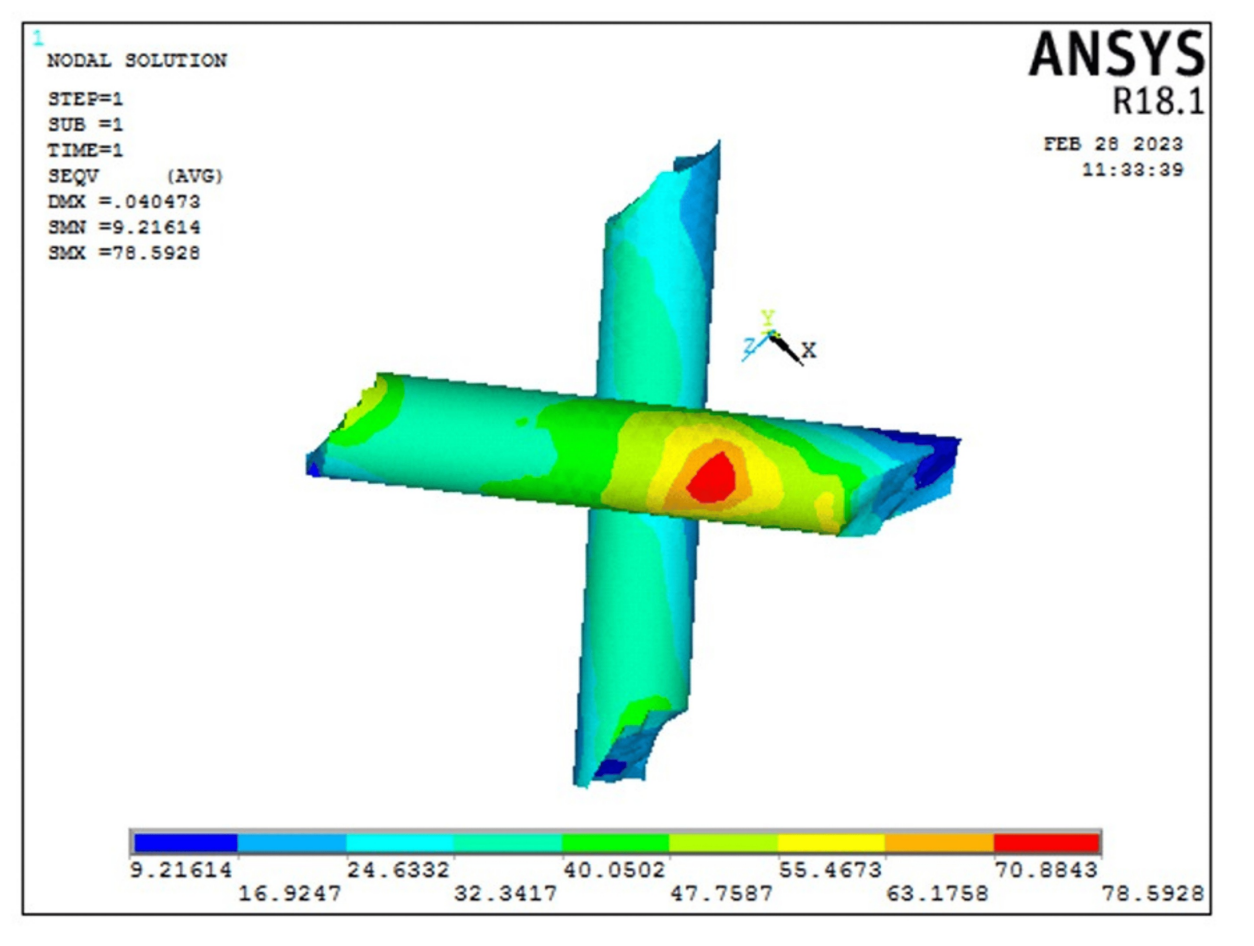
von Mises stress on the transverse fiber post used for restoration. Note that the area of stress concentration is within the center of the cross section of both posts away from the tooth structure. The color bar is used to visualize the results of structural analysis by representing the intensity of mechanical stress, with warmer colors indicating high-stress areas and cooler colors indicating low-stress areas

The peri stress of Model 1 representing the intact model (10.083 Mpa) was less as compared to that seen in Model 2 representing the transverse fiber post model (10.2327 MPa). Additionally, in the transverse fiber post model, fiber post stress was 78.59 MPa and composite stress was 75.69 MPa. The stress on the transverse fiber post is represented in Figure 7.

The table comparing all the abovementioned stress and displacement figure is given as Table 2. 

**Table 2:** Comparison of values representing von Mises stresses on both the intact tooth model and experimental tooth model

Description	Intact model	Experimental model
Overall deformation	0.046879	0.050627
Overall stress (Mpa)	154.83	119.294
Cortical stress (Mpa)	17.06	17.7028
Cancellous stress (Mpa)	9.66112	8.96254
Dentine stress (Mpa)	80.7399	78.0512
Enamel stress (Mpa)	154.83	119.294
Fiber stress	-	10.2327
Peri stress (Mpa)	10.083	0.000000525
Pulp stress (Mpa)	0.000000542	78.5928

The functional cusp of intact mandibular molars exhibited the largest von Mises stress concentration, as revealed by the stress analysis. For the intact mandibular molar with MOD cavity, the analysis revealed that the major concentration of the forces acting was taken up by the functional cups of the tooth. When comparing the intact tooth model and the restored transverse tooth model, it was seen that there was an overlap of the concentrated stress in the functional cusp region in both models. Of special note is that the concentration of stress reflected on the fiber post was in the region of overlap in the midsection of the post, which had no bearing on the interface of restoration and the tooth structure. 

**Figure 8:**
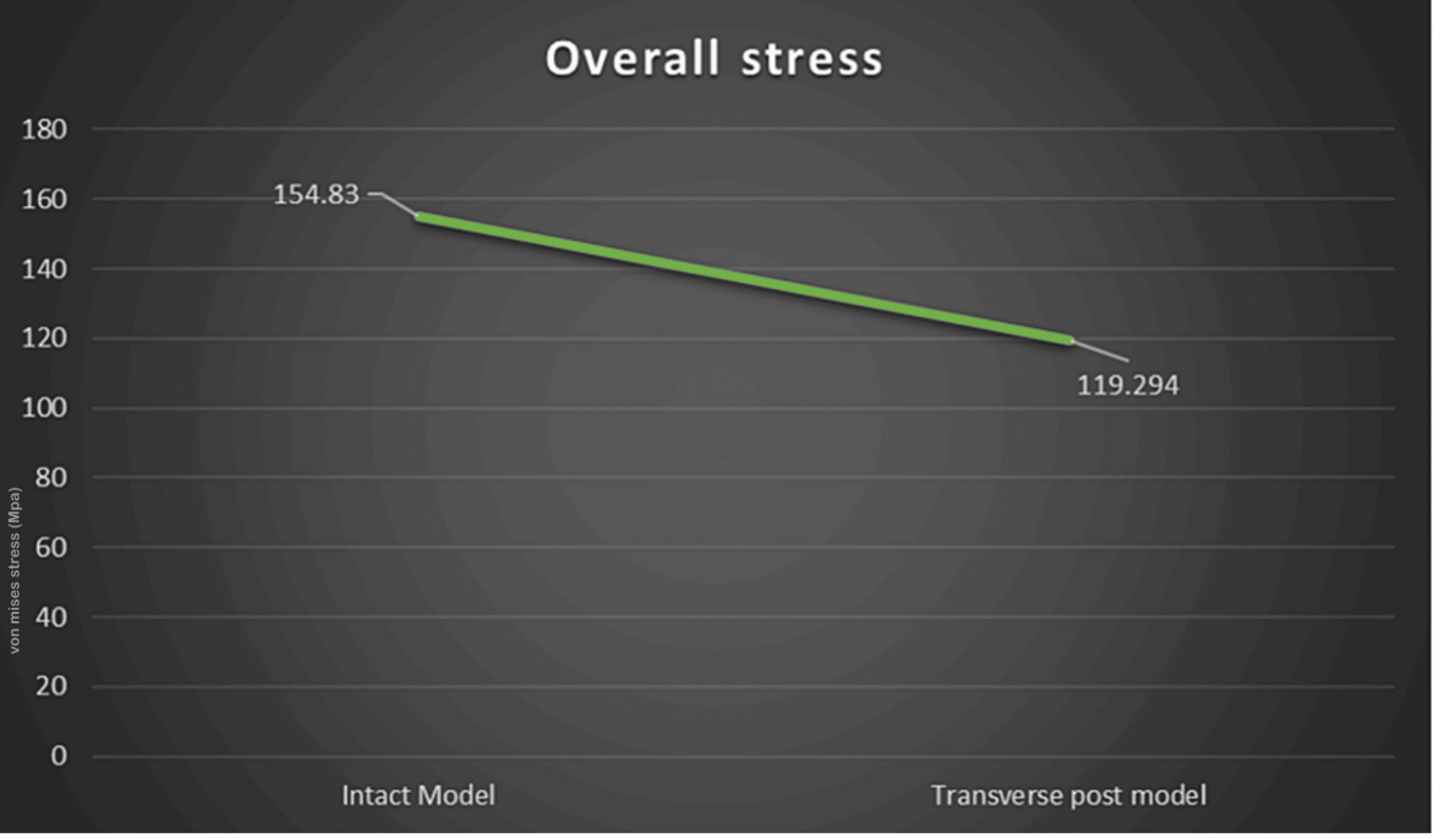
Graphical representation of overall stress in both Model 1 and Model 2

Stress maps for the restorative interfaces, dentin tissue, and enamel tissue were used to summarize the von Mises stress data. Stress levels from the MOD cavity model and the intact tooth model were found to differ significantly. Using the same data, a nonparametric test was used to confirm the findings. The Mann-Whitney U test was undertaken. The p-value was 0.02 which suggested clinical significance. Figure 8 shows the line graph representation of all the abovementioned data in regard to the overall deformation under the functional occlusal forces as seen in both Model 1 and Model 2.

**Figure 9:**
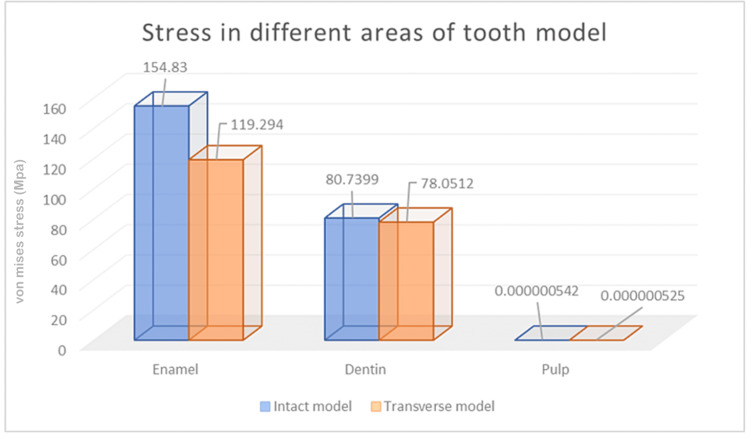
Graphical representation comparing the von Mises stress distribution on enamel, dentine, and pulp between Model 1 (intact model) and Model 2 (transverse fiber post restored model)

Figure 9 shows the graphical representation of all the abovementioned data in regard to deformation in enamel, dentin, and pulp due to the functional occlusal forces as seen in both Model 1 and Model 2.

## Discussion

In recent years, various developments in the field of restorative dentistry have emerged in order to improve upon the properties of restorative materials to resemble the intact tooth structure in function and aesthetics. The incorporation of fibers helps in stress distribution in prepared non-vital teeth essentially reinforcing the tooth structure. The incorporation of fibers in restorative materials has broadened the horizons for direct restorations. Short fiber-reinforced composite (SFRC) materials are particularly beneficial in replacing natural tooth structure in large defects, due to the deflection of stresses. In such materials, reinforcement occurs in three directions with fibers distributed randomly. On the other hand, woven continuous and bidirectional fibers offer bidirectional augmentation, but they are stronger compared to SFRC materials. Leno woven ultra-high-molecular-weight (LWUHMW) polyethylene fiber ribbons (e.g., Ribbond THM) and bidirectional fiber-reinforced composite (FRC) (such as EverStick Net) are being utilized in various restorations [19,20]. The secondary function of their stress-absorbing capacity during the healing process is to act as a splint that enhances resistance against fracture propagation [21].

For class II MOD cavity restorations, the FEA approach was selected as it is instrumental in visualizing the distribution of stress in various restorations. In a previously done study, Ausiello et al. did not consider the material incremental thickness in their investigation, while in our model, simulation with the material thickness presents results parallel with the previous study [22]. von Mises stress is a common value used to represent data from FEA. By integrating the three main stresses, tensile, compressive, and shear, von Mises stress is an entity that highlights those sections of the model that are most likely to fail when subjected to various levels of stress. Furthermore, it is acknowledged that the greatest primary stress is a useful metric for assessing the failure of a presumed brittle material.

In a similar study done by Alp et al., for every restoration model that was examined, the results obtained from the intact model revealed noticeably inferior values for the dentin and enamel structure [23]. In the present study, the model restored with the transverse post showed a similar if not better distribution of stress than that seen in an intact tooth model for all components: enamel, dentin, and pulpal regions. This correlates clinically to the restoration of the MOD cavity with transverse post and composite as being a post-endodontic treatment modality as there are promising results with stress distribution. Similarly, in a study done by Sharma et al. that involved an intact model and a model with horizontal posts of three different thicknesses, the overall stress was higher in fiber posts of 1 mm and 1.5 mm width but was less in the one with 2 mm width when compared to an intact tooth model. In the present study, the MOD cavity presenting in a permanent molar was assessed. This is in line with previous studies that examined stress distribution based on various cavity preparations with varying C factors [24,25].

In the present study, there is only one cavity design and one restoration which was assessed. This is a huge limitation of the study, and further studies have to be conducted to corroborate the same. According to Yang et al., the highest von Mises stress was found in teeth treated with resin composite, followed by zirconia and then glass ceramic. A zirconia inlay for the restoration of a MOD cavity showed that interfacial shear stresses dropped when the isthmus width was increased, but the stress levels remained unchanged for all three materials when the prepared cavity depth increased [26].

This study has a few limitations. The main limitation is that only one model of the MOD cavity was compared to the intact tooth model. Only one arrangement of transverse fibers was assessed in the study. The conditions replicated represented all the physical aspects of the materials and tooth included, but not that seen in the oral cavity. Hence, there should be more studies comparing different tooth cavity designs and different restorative materials in a clinical setup.

## Conclusions

The conservative restoration of MOD cavities especially in non-vital teeth poses serious problems as full coverage crowns are always preferred. Within the limitations of this study, the stress distribution due to the rehabilitation of a non-vital permanent mandibular molar presented with MOD cavity preparation, using a 2 mm horizontal post in a transverse fashion, was comparable to that of an intact tooth in terms of stress distribution. Thus, this suggests that the transverse horizontal posts as a framework along with composite restoration can be used as a viable treatment modality for a sound and functional, post-endodontic restoration in permanent molars.
